# Inverted temporal internal limiting membrane flap technique for chronic large traumatic macular hole

**DOI:** 10.3205/oc000154

**Published:** 2020-05-28

**Authors:** Archana Kumari, Lalit Agarwal, Nisha Agrawal, Sabin Sahu, Indranath Prasad, Deepti Pradhan

**Affiliations:** 1 Biratnagar Eye Hospital, Biratnagar, Nepal; 2 Taparia Eye Care, Biratnagar, Nepal; 3 Sagarmatha Choudhary Eye Hospital, Lahan, Nepal; 4 Prasad Eye Hospital, Ramgarh, India

**Keywords:** traumatic macular hole, chronic large traumatic macular hole, inverted temporal internal limiting membrane flap technique

## Abstract

Various modifications of surgical techniques and surgical adjuncts are adopted with standard pars plana vitrectomy (PPV) to improve the outcome of traumatic macular hole (TMH) surgeries. We describe a successful closure of a chronic large TMH of three years duration with inverted temporal internal limiting membrane (ILM) flap technique. A 36-year-old male patient had an optical coherence tomography (OCT) documented chronic macular hole (MH) for three years following blunt trauma. Fundus examination also showed choroidal rupture scar temporal to fovea. The minimum MH diameter was 769 µ and the basal diameter 1431 µ in OCT. Standard PPV with inverted temporal ILM flap and gas tamponade was done. The postoperative period was uneventful. The best corrected visual acuity improved from 6/60 preoperatively to 6/18 six months postoperatively, and OCT showed a closed MH with anatomical type 1 closure. This case highlights that the inverted temporal ILM flap technique is a safe and effective technique for patients with even chronic and large TMH.

## Introduction

The incidence of traumatic macular holes (TMH) is relatively rare compared to their idiopathic counterparts. It is reported to occur in 1.4% of closed globe injuries and 0.15% of open globe injuries [1]. TMH have long been thought to arise from a contrecoup mechanism, and are likely supplemented by varying degrees of vitreous traction within the globe [2]. A sudden decrease in the globe’s anterior-posterior diameter causes a compensatory equatorial expansion. This dynamic change within the volume-fixed globe can lead to horizontal forces and splitting of the retinal layers at the fovea. Unlike the slowly evolving vitreoretinal traction of an idiopathic macular hole (IMH), the tangential vitreous forces created by blunt ocular trauma are more variable. This variation can often lead to a much more irregular configuration of the hole than the often perfectly circular shape seen in IMH [2]. Also due to the individual uncertainty of the force imposed on the eye and the inherent structural features of the eye, the extent of the retinal injury and the progression of TMH are still difficult to predict clinically [3].

Spontaneous closure of small-size TMH is shown to occur in about two-thirds of the reported cases within up to three months of trauma, and nearly all holes closed within six months with significant visual improvement [3]. However, observation for spontaneous closure is recommended for young patients with small holes, good visual acuity, and posterior vitreous adhesion to the hole edges [3]. Despite the varying contributions of vitreous traction to its pathogenesis, the current surgical techniques of vitreous surgery for TMH are similar to those for IMH, including removal of the posterior hyaloid, epiretinal membranes (if present), with or without internal limiting membrane peeling (ILMP), and intraocular gas or silicone oil tamponade. However, traditional pars plana vitrectomy (PPV) with ILMP and tamponade has poorer outcome in TMH compared to IMH. This led to trial of other modifications of ILMP.

Recently, inverted internal limiting membrane (ILM) flap technique with favorable anatomic and functional outcome for large IMH was introduced [4], [5], [6], [7], [8], [9], [10]. The inverted ILM flap may induce glial cell proliferation, resulting in the macular hole (MH) filling with proliferating cells that enhance closure. It may also act as a scaffold for cell proliferation to promote the closure of the MH [4]. We operated on a case of chronic large TMH of three years duration with the inverted temporal ILM flap technique and observed excellent anatomical and functional results.

## Case description

A 36-year-old male patient presented with a chronic MH (with optical coherence tomography [OCT] documentation for 3 years) following blunt trauma with a cricket ball 6 years earlier.

**Figure 1:**
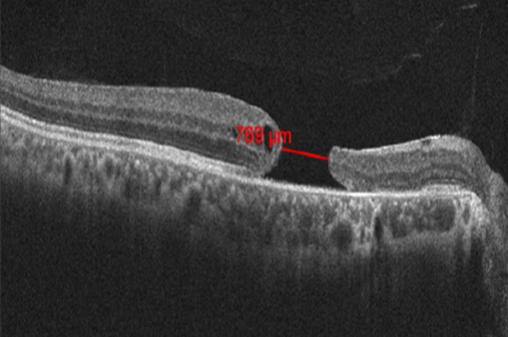
Preoperative optical coherence tomography showing full thickness traumatic macular hole. The minimum linear diameter was 769 µm.

On examination, his best corrected visual acuity (BCVA) was 6/60 in the right eye and 6/6 in the left eye. Intraocular pressure was normal in both eyes. Anterior segment examination results were within normal limits. Fundus examination of the right eye showed a normal optic disc and a large full thickness TMH with no sign of posterior vitreous detachment. Choroidal rupture scar was seen about 3 disc diameters temporal to the fovea and a pigment clump was seen superotemporal to the disc. OCT measured the minimum MH diameter as 769 µ, the basal diameter 1431 µ and height 272 µ. Intraretinal cystic spaces were present on the temporal side of the MH (Figure 1 (Fig. 1)).

The patient underwent PPV with inverted temporal ILM flap technique and 14% perfluoropropane (C3F8) tamponade. After 23G PPV, ILM was stained with 0.05% brilliant blue G. A semicircular ILM peeling was carried out about 2 disc diameters above the fovea extending circumferentially via temporal approach to end just below the macula. ILM peel was done just up to the temporal edge of the hole and the flap was inverted and gently coaxed over the MH. Fluid-gas exchange was promptly done and the vitreous cavity filled with non-expansile concentration of 14% C3F8. The patient was advised to remain in the face-down position for one week. The postoperative period was uneventful.

**Figure 2:**
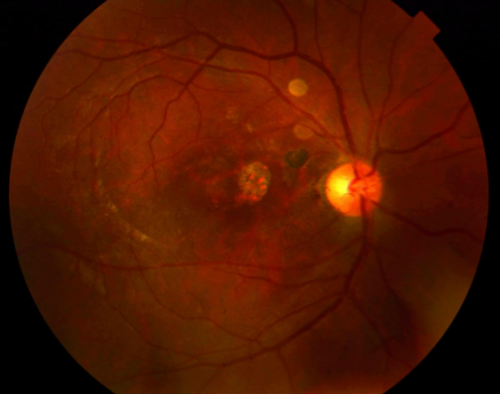
Fundus photo 6 months postoperative showing closed macular hole

**Figure 3:**
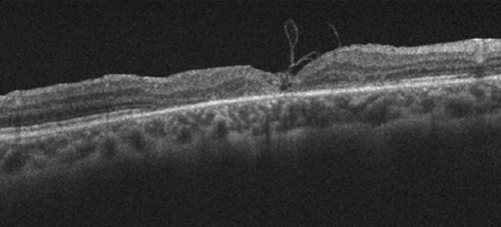
Optical coherence tomography 6 months postoperative showing anatomical type 1 closure of the macular hole

Two weeks after the surgery, the visual acuity improved to 6/36 and OCT showed a gradual decrease in size of the MH. Six months postoperatively, the patient’s BCVA improved to 6/18, closure of TMH was seen clinically (Figure 2 (Fig. 2)), and OCT showed type 1 anatomical closure (Figure 3 (Fig. 3)).

## Discussion

TMH are well-known complications following ocular trauma. The visual consequences of TMH and their associated injuries can be severe. The modern advancements in techniques and equipments in vitreoretinal surgery have significantly improved the anatomical and functional outcome of MH surgeries.

A lot of surgical adjuncts have been adopted in addition to standard vitrectomy, including the use of serum transforming growth factor β2, platelets, autologous serum, and plasmin to improve the rate of TMH closure [2], [3]. Among all the different techniques for MH surgery, the one with the most positive effect on final outcome is PPV with ILM peeling, to release tangential forces acting on the MH. The peeled-off ILM itself contains Müller cell fragments; therefore, ILM peeling alone can induce gliosis. Thus, if a segment of peeled-off ILM is left attached, it may provoke gliosis both inside the retina and on the surface of the ILM. The ILM also may be a scaffold for tissue proliferation [4].

Abou Shousha [5] assessed the role of inverted ILM flap as a treatment option for large TMH. In a prospective non-comparative study of 12 eyes with large TMH (basal diameter of 1300–2800 µm), a 100% closure rate and improvement of BCVA were achieved 6 to 9 months after the surgery [5]. Inverted ILM flap technique is shown to have high incidence of type 1 closure with improved functional outcome in large MH as compared to standard ILM peel [6].

The temporal inverted ILM flap technique is reported to be advantageous as the spontaneous detachment or flip back of the flap does not occur, and is especially useful in patients who are unable to maintain postoperative prone positioning [9]. Temporal inverted ILM flap technique is demonstrated to produce similar closure rates and visual acuity improvements for large full thickness macular holes (FTMH) compared to the original inverted ILM flap technique [10]. Additionally, the temporal inverted ILM flap technique in which the ILM is removed only from the temporal side of the MH tended to be associated with fewer cases of dissociated optic nerve fibre layer (DONFL) defect simultaneously preserving the papillomacular bundle from iatrogenic trauma [10].

In our case, a large chronic TMH was associated with a choroidal rupture scar temporally, so we decided to do the inverted temporal ILM flap technique and obtained an excellent anatomical and functional outcome. 

## Conclusion

This report highlights that the temporal inverted flap technique is a safe and effective option for patients with large and chronic TMH. However, further study with larger samples will be helpful to confirm the advantages of this technique over other techniques.

## Notes

### Abbreviations

- TMH: traumatic macular hole
- IMH: idiopathic macular hole
- FTMH: full thickness macular hole
- ILM: internal limiting membrane
- ILMP: internal limiting membrane peeling
- PPV: pars plana vitrectomy
- OCT: optical coherence tomography
- DONFL: dissociated optic nerve fibre layer

### Competing interests

The authors declare that they have no competing interests.
